# Transcriptome changes in DM1 patients’ tissues are governed by the RNA interference pathway

**DOI:** 10.3389/fmolb.2022.955753

**Published:** 2022-08-19

**Authors:** Maya Braun, Shachar Shoshani, Yuval Tabach

**Affiliations:** Tabach Laboratory, Department of Developmental Biology and Cancer Research, Institute for Medical Research Israel-Canada, Hebrew University of Jerusalem, Jerusalem, Israel

**Keywords:** small RNAs, RNA interference, myotonic dystrophy type 1, expansion repeat disorders, trinucleotide repeats, gene signature

## Abstract

Myotonic dystrophy type 1 (DM1) is a multisystemic disease caused by pathogenic expansions of CTG repeats. The expanded repeats are transcribed to long RNA and induce cellular toxicity. Recent studies suggest that the CUG repeats are processed by the RNA interference (RNAi) pathway to generate small interfering repeated RNA (siRNA). However, the effects of the CTG repeat-derived siRNAs remain unclear. We hypothesize that the RNAi machinery in DM1 patients generates distinct gene expression patterns that determine the disease phenotype in the individual patient. The abundance of genes with complementary repeats that are targeted by siRNAs in each tissue determines the way that the tissue is affected in DM1. We integrated and analyzed published transcriptome data from muscle, heart, and brain biopsies of DM1 patients, and revealed shared, characteristic changes that correlated with disease phenotype. These signatures are overrepresented by genes and transcription factors bearing endogenous CTG/CAG repeats and are governed by aberrant activity of the RNAi machinery, miRNAs, and a specific gain-of-function of the CTG repeats. Computational analysis of the DM1 transcriptome enhances our understanding of the complex pathophysiology of the disease and may reveal a path for cure.

## Introduction

Thousands of repetitive DNA sequences are scattered across the human genome in coding and non-coding regions (Pelley, 2007). Expansions of some of these repeats, such as CTG, CGG, and CCTG, are the source of over 40 genetic diseases (Ellerby, 2019). Repeats that occur in coding regions of the genome lead to protein-based disorders [reviewed by Paulson (Paulson, 2018)], such as Huntington’s Disease. Other types of repeats that occur in non-coding regions are transcribed to RNA and form imperfect hairpins structures that can become toxic to the cell (termed RNA toxicity), although only rarely translated (Marquis Gacy et al., 1995; Darlow and Leach, 1998; Tam et al., 2003; Dere et al., 2004; Liu et al., 2010; Zu et al., 2011). Interestingly, an expansion of a specific sequence from different loci and different genes can trigger similar disease phenotypes. For example, spinocerebellar ataxias are caused by CAG repeat expansions in thirteen different genes, and familial adult myoclonic epilepsy was linked to (TTTTA)_n_(TTTCA)_m_ repeats in five separate genes (Ishiura et al., 2018; Corbett et al., 2019; Florian et al., 2019; Zeng et al., 2019). This implies a common molecular mechanism for RNA toxicity that results from the repeats even under different genetic contexts.

Myotonic dystrophy type 1 (DM1) is a multisystemic disease caused by an expansion of the CTG repeat sequences in the 3′UTR of the DMPK gene (Brook et al., 1992; Fu et al., 1992; Mahadevan et al., 1992). The long RNA repeats fold to create hairpin structures and accumulate as RNA foci (Davis et al., 1997). The expanded sequence exhibits tissue-specific repeat instability, particularly in striated muscle, and may further lengthen through life (López Castel et al., 2010). Although the most prominent symptoms of DM1 are progressive muscle weakness and myotonia, different tissues exhibit distinct pathologies (Harper, 2009). Patients typically suffer from cardiac abnormalities, gastrointestinal symptoms, early-onset cataracts, premature frontal balding, sleep disturbance, and more. MBNL proteins, a family of RNA-binding splicing factors, preferentially bind to double-stranded CUG RNAs, in proportion to the length of the hairpin (Miller, 2000). Therefore, in DM1, they are sequestered by the RNA foci and their activity is diminished (Philips et al., 1998; Kuyumcu-Martinez et al., 2007; Laurent et al., 2012; Nakamori et al., 2013). However, transcriptional alterations caused by splicing disruption explain only some of the disease manifestations (Savkur et al., 2001; Charlet. et al., 2002; Mankodi et al., 2002; Fugier et al., 2011). Additional mechanisms such as microRNA dysregulation (Fernandez-Costa et al., 2013) and repeat associated non-ATG translation (Zu et al., 2011) have been demonstrated. Furthermore, DM1 patients show global transcriptomic changes across tissues and along the disease’s progression. Further understanding of these changes can shed light on the disease mechanism and progression. Specifically, it is unclear why certain genes are up or downregulated in different patients, or across tissues, or which or why certain transcription factors and miRNAs are involved in the process.

Numerous studies have shown that expanded CTG repeats trigger disease phenotypes which recapitulate DM1 symptoms and alter gene expression across species (de Haro et al., 2006; Qawasmi et al., 2019). In prior work performed in *C. elegans,* we revealed that the RNA interference (RNAi) pathway is an important player in mediating these changes (Qawasmi et al., 2019). Dicer targets and slices the transcribed CUG repeats into small repeated RNAs, which then bind and silence genes with complementary sequences, inducing transcriptomic changes. This is supported by previous research which showed that Dicer processes the repeats to siRNA in DM1 patient-derived cells (Krol et al., 2007). Here, we aimed to investigate the DM1 transcriptome and the regulatory network shaping it. We hypothesized that in patients, as in model organisms, the RNAi pathway, in addition to other factors, plays an important role in perturbating gene expression. RNA interference, triggered by repeat-derived siRNAs, mediates the silencing of genes that carry endogenous CTG/CAG repeats. By integrating transcriptomic analyses of published RNA-seq data from DM1 patient biopsies we were able to demonstrate characteristic changes in gene expression. The transcriptional alterations can mechanistically link the RNAi machinery and the CTG repeats and point at distinct DM1 transcriptomic subtypes.

## Material and methods

### Data resources

Expression profiling by high throughput sequencing was obtained from three published cohorts. The datasets contained tibialis samples of 40 adult DM1 patients and 10 unaffected controls (Wang et al., 2019), nine heart autopsy samples from DM1 patients and three healthy controls, biceps branchii autopsy tissue of three congenital DM1 infants and three disease controls (SMA type 1) (Thomas et al., 2017), and adult frontal cortex samples from post-mortem frozen brains of 21 DM1 patients and eight unaffected controls (Otero et al., 2021).

### Differential expression analysis

Raw RNA-seq data was downloaded from the GEO database and quantified to gene counts through the ARCHS4 pipeline (Lachmann et al., 2018), using BioJupies (Torre et al., 2018). The sequencing data were uploaded to the Galaxy web platform, and we used the public server at usegalaxy.org to analyze the data (Afgan et al., 2018). The identification of differentially expressed genes was performed using the limma-voom package (Smyth, 2005; Ritchie et al., 2006; Law et al., 2014; Liu et al., 2015). The samples were normalized using the TMM method. Genes without more than 1CPM in all the samples for each condition were considered insignificant and filtered out for low expression. Benjamini–Hochberg false discovery rate (FDR) method was applied and adjusted *p*-values of <0.05 were considered statistically significant. Hierarchical clustering was performed using the UPGMA method (Bar-Joseph et al., 2001). The heatmaps were established using the matplotlib (Hunter, 2007) and seaborn (Waskom, 2012) packages in Python.

### Identification of genes with endogenous trinucleotide repeats

A BLAST search was performed to identify genes bearing four to seven repeats of CTG/CAG in the RefSeq Select RNA sequence (Altschul et al., 1990). Replicate genes were removed resulting in 2,387 genes bearing 4CTG/CAG repeats, 1,186 genes with 5CTG/CAG repeats, 985 genes containing 6CTG/CAG repeats with two or less mismatches, and 678 genes bearing 7CTG/CAG repeats with two or less mismatches. The same process was performed to identify genes bearing other pathogenic trinucleotide repeats, resulting in 481 genes with 6CGG/CCG repeats, 73 genes with 6CGT/ACG repeats, 331 genes with 6GAA/TTC repeats, and 710 genes with 6GCG/CGC repeats. Significance for enrichment of repeat bearing genes was determined using Fisher’s exact test.

### Correlations between muscle impairment and transcriptomic alterations

The correlation between expression level values of genes bearing 6CTG/CAG repeats in each patient and their normalized dorsiflexion strength score [obtained as previously described (Wang et al., 2019)] was calculated. The Pearson’s correlation coefficient was computed using the python SciPy library (Virtanen et al., 2020). Principal component analysis (PCA) was performed using the normalized expression levels of all genes. The first two principal components were plotted.

### Enrichment analysis

To identify enriched biological pathways, gene ontology analysis was performed using Enrichr (Chen et al., 2013; Kuleshov et al., 2016b; Xie et al., 2021) based on Reactome gene sets (Croft et al., 2014). Transcription factor enrichment analysis was performed using ChEA3 (Keenan et al., 2019). As siRNAs average in length of 18 base pairs, we intersected and identified the transcription factors that carry six consecutive CTG/CAG repeats. We applied Kolmogorov-Smirnov tests to evaluate the distribution of the ranking of the repeat-bearing TFs among all the TFs that were significantly enriched for the deregulated genes in DM1 patients. Gene set enrichment analysis for the regulatory miRNA pathways was performed using GSEA (Mootha et al., 2003; Subramanian et al., 2005).

### System expression analysis

An enrichment analysis using GeneAnalytics (Fuchs et al., 2016) was performed to identify the expression of repeat-carrying genes in various systems. Systems were defined by LifeMap (Edgar et al., 2013) as a collection of organs, which share similar physiological functions. The system score is calculated as a weighted sum of the scores of all the matched genes in this system, normalized to the log of the maximal score of the system. Statistical significance was calculated by Fischer’s exact test.

## Results

### Gene expression pattern predicts muscle impairment in DM1 patients

To better understand the patients’ transcriptome and its correlation with the pathophysiology of DM1, we wish to reveal which genes are up or down regulated, if patients can be divided to subgroups, and whether the transcriptome can be associated with muscle impairment in patients. Hence, we analyzed RNA-seq data of tibialis muscle biopsies taken from 40 DM1 patients and 10 healthy individuals (Wang et al., 2019). Weakness of distal muscles, especially dorsiflexors, is a characteristic early symptom of DM1 (Hiba et al., 2012; Bouchard et al., 2015). The tibialis anterior muscle is affected at both functional and histological level at an early stage of the disease, thus marking it most suitable for transcriptome analysis in DM1. Measurements of ankle dorsiflexion strength were also available for these patients, reflecting the severity of muscle impairment.

**FIGURE 1:**
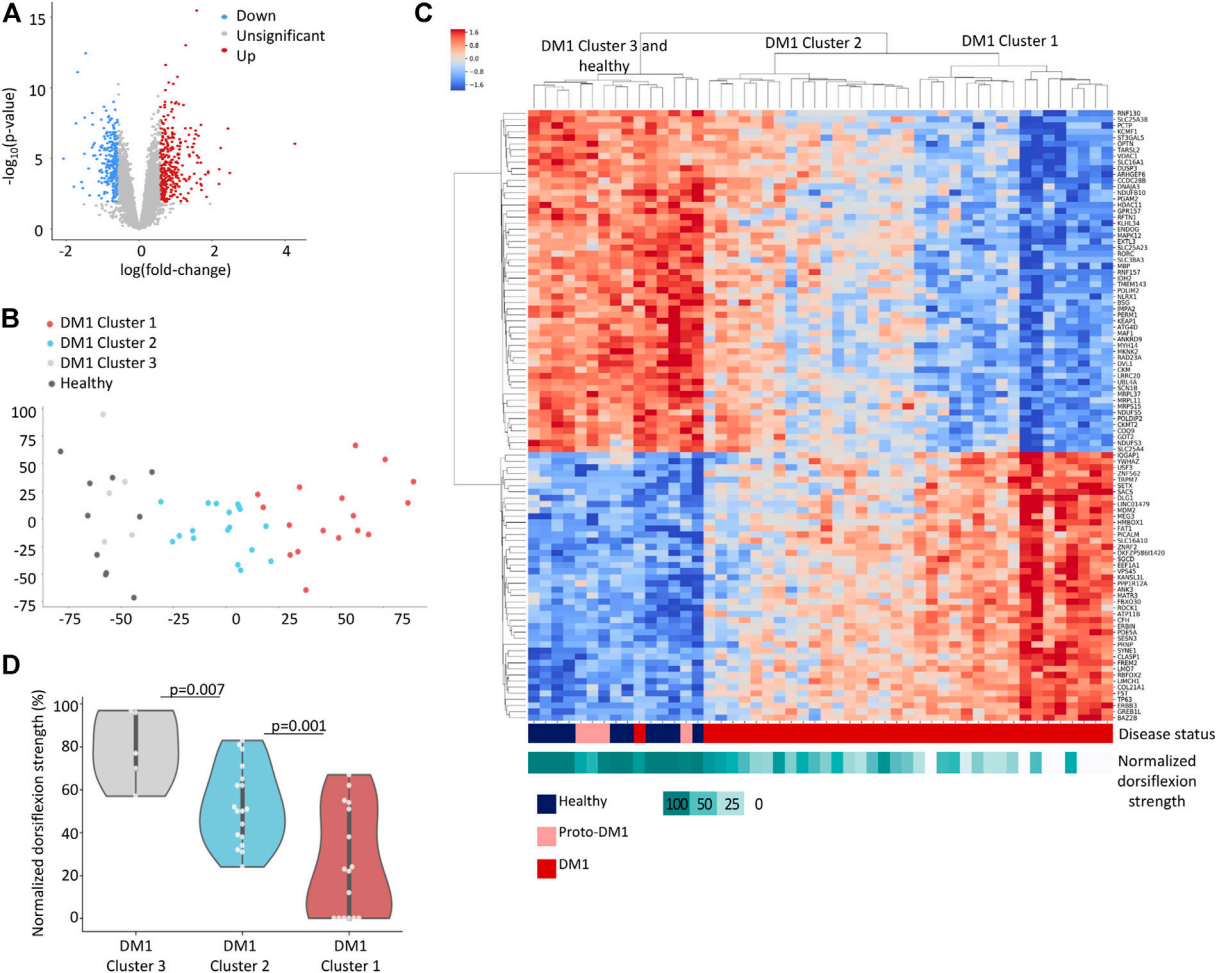
DM1 gene expression signature. **(A)** Volcano plot displaying differentially expressed genes between 40 DM1 patients and 10 healthy individuals. 1.5-fold change upregulated genes are highlighted in red, 1.5-fold change downregulated genes are highlighted in blue. **(B)** PCA analysis of normalized gene expression values for Tibialis DM1 and healthy samples. **(C)** Heatmap of normalized expression levels of [top 100 loading score (Jolliffe, 2011)] genes across Tibialis samples from 40 DM1 patients and 10 healthy individuals. The rows and columns are ordered based on hierarchical clustering (by the UPGMA method). Each row represents a single gene, and each column represents a sample. The colors indicate the normalized expression levels from high (red) to low (blue). The normalized dorsiflexion strength score of each individual is depicted in the bottom bar, ranging from white (lowest score) to cyan (highest score). **(D)** Normalized dorsiflexion strength scores of healthy individuals and DM1 patients, grouped according to the clusters formed in Figure 1C.

We preprocessed and filtered the gene expression data (see Methods). Overall, 1,565 genes were significantly over-expressed and 1,664 genes were under-expressed in DM1 patients compared to healthy individuals [FDR adjusted *p*-value <0.05, using the limma-voom method (Law et al., 2014)] (Figure 1A; Supplementary Table S1). To reduce noise, we filtered out genes with less than 1.5-fold-change and identified 319 upregulated genes and 275 downregulated genes in DM1 (highlighted in red and blue, respectively). We analyzed the upregulated genes [using enrichR (Kuleshov et al., 2016a) on the Reactome (Croft et al., 2014; Fabregat et al., 2017) database] and found that they are highly enriched for collagen biosynthesis (FDR = 4.5 × 10^−9^), extracellular matrix organization (FDR = 7.3 × 10^−12^) and striated muscle contraction (FDR = 0.015), (Table 1). These pathways are associated with typical disease symptoms such as myotonia and fibrosis observed at end-stage disease (Gagnon et al., 2010; Meola and Cardani, 2015; De Antonio et al., 2016). In contrary, the downregulated genes show limited association with general biological functions (i.e., citric acid cycle, glucose, creatine, and pyruvate metabolism) that are associated with a wide variety of conditions.

**TABLE 1:** Reactome pathways enriched for the differentially expressed genes in DM1. FDR-adjusted *p*-values are presented.

Term	FDR *p*-value
Upregulated genes in DM1	
Extracellular matrix organization *Homo sapiens* R-HSA-1474244	7.31E-12
Collagen biosynthesis and modifying enzymes *H. sapiens* R-HSA-1650814	4.53E-09
Assembly of collagen fibrils and other multimeric structures *H. sapiens* R-HSA-2022090	1.17E-07
Collagen formation *H. sapiens* R-HSA-1474290	1.17E-07
ECM proteoglycans *H. sapiens* R-HSA-3000178	0.002702
Striated muscle contraction *H. sapiens* R-HSA-390522	0.015191
Scavenging by class A receptors *H. sapiens* R-HSA-3000480	0.015191
NCAM1 interactions *H. sapiens* R-HSA-419037	0.018716
Integrin cell surface interactions *H. sapiens* R-HSA-216083	0.034147
Diseases associated with glycosaminoglycan metabolism *H. sapiens* R-HSA-3560782	0.034147
A tetrasaccharide linker sequence is required for GAG synthesis *H. sapiens* R-HSA-1971475	0.034147
Presynaptic nicotinic acetylcholine receptors *H. sapiens* R-HSA-622323	0.03457
Muscle contraction *H. sapiens* R-HSA-397014	0.041894
Activation of nicotinic acetylcholine receptors *H. sapiens* R-HSA-629602	0.041894
Postsynaptic nicotinic acetylcholine receptors *H. sapiens* R-HSA-622327	0.041894
Acetylcholine binding and downstream events *H. sapiens* R-HSA-181431	0.041894
Downregulated genes in DM1	
Metabolism *H. sapiens* R-HSA-1430728	1.16E-04
Pyruvate metabolism *H. sapiens* R-HSA-70268	3.89E-04
The citric acid (TCA) cycle and respiratory electron transport *H. sapiens* R-HSA-1428517	0.001577
Pyruvate metabolism and citric acid (TCA) cycle *H. sapiens* R-HSA-71406	0.005217
Proton-coupled monocarboxylate transport *H. sapiens* R-HSA-433692	0.005217
Gluconeogenesis *H. sapiens* R-HSA-70263	0.005261
Glycolysis *H. sapiens* R-HSA-70171	0.005261
Glucose metabolism *H. sapiens* R-HSA-70326	0.007012

To uncover complex gene expression patterns and link expression with disease progression we conducted an unsupervised analysis. We applied principal component analysis (Figure 1B) and Hierarchical clustering (Figure 1C) to cluster the genes and patients. While unaffected individuals clustered together, the DM1 patients showed diverse gene expression and clustered into three major groups that display a trend of worse muscle impairment along the first principal component (Figures 1B–D). Notably, four out of the five DM1 patients that cluster with the healthy controls carry proto-DM1 mutations (less than 100 CTG repeats). These results correlate the patient’s disease state (e.g., expansion length) and global gene expression. This might point to disruption in transcriptional regulation of the genes.

### Genes bearing endogenous CTG/CAG-repeats are differentially expressed between DM1 patients and controls

**FIGURE 2:**
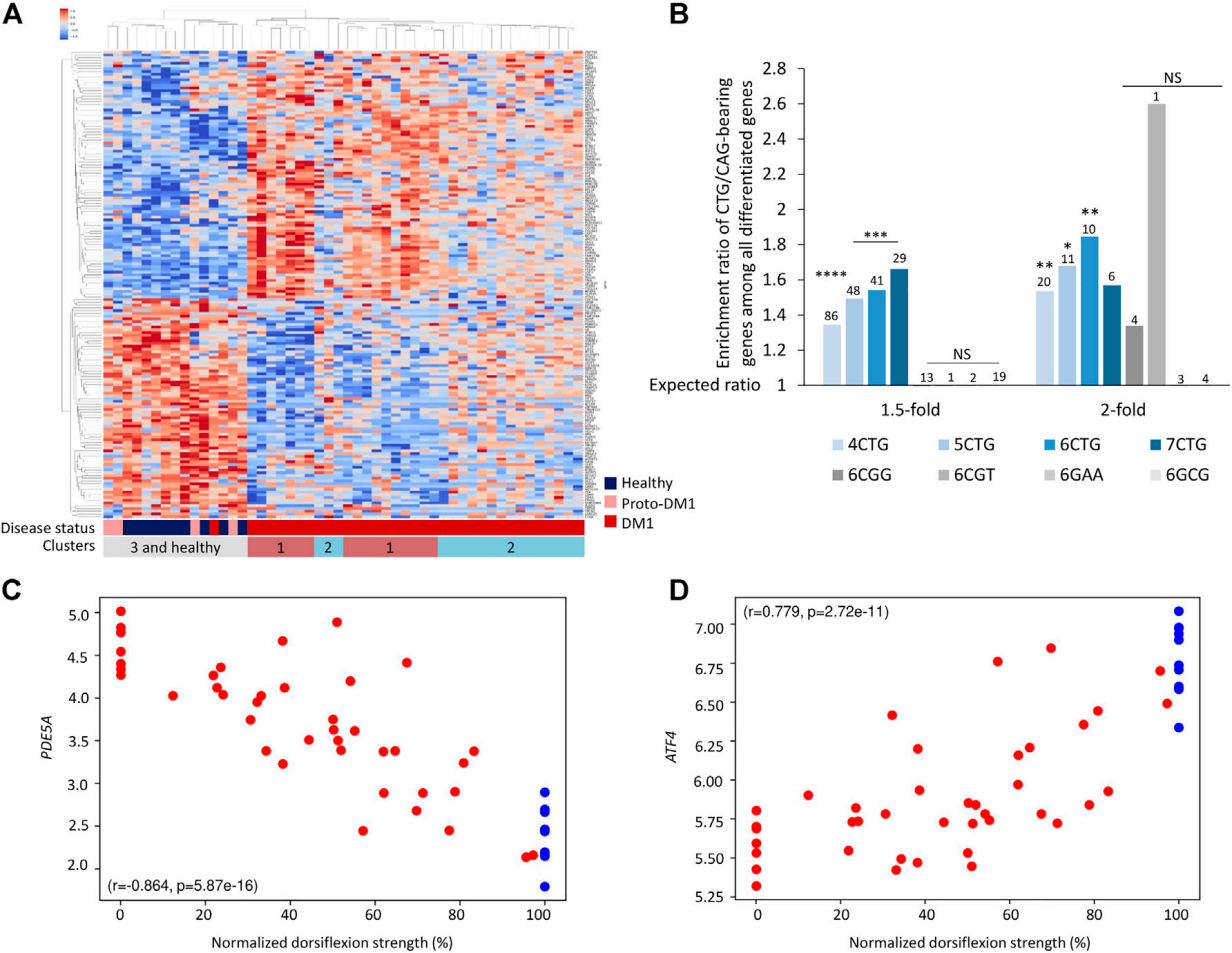
Genes bearing endogenous CTG/CAG -repeats are differentially expressed between DM1 patients and controls. **(A)** Heatmap of normalized expression levels of differentially expressed genes bearing endogenous 6CTG/CAG repeats across Tibialis samples from 40 DM1 patients and 10 healthy controls. **(B)** Bar plot presents ratio of genes bearing CTG/CAG repeats that are differentially expressed in DM1 samples divided by the expected number derived from the prevalence of those genes in the tissue. Different fold-change thresholds and lengths of consecutive CTG/CAG repeats are presented. Additional combinations of pathogenic repeats (6CGG, 6CGT, 6GAA, and 6GCG) are depicted in gray. The number of genes differentially expressed for each group is indicated above the corresponding bar. Bars with at least two differentially expressed genes are presented. **(C,D)** Expression levels of top-ranking differentially expressed genes bearing 6CTG/CAG repeats relative to normalized dorsiflexion strength of 40 DM1 patients and 10 healthy individuals. Pearson’s correlation coefficient (*r*) and *p*-value (*p*) are depicted. *0.05; **0.01; ****0.0001.

Next, we aim to understand which regulatory components might explain the changes in gene expression, and specifically if some of these changes can be linked to the RNAi machinery. We previously showed in an animal model that the expanded CUG repeats are processed by the RNAi machinery to short-interfering RNAs and silence targeted genes (Qawasmi et al., 2019; Braun et al., 2022). The expanded CTG repeats are transcribed bidirectionally, creating both CAG(n) and CUG(n) RNAs (Michel et al., 2015; Gudde et al., 2017). siRNAs average in length of ∼18 nucleotides. Hence, we considered genes with 6 CUG/CAG repeats as the most likely targets. Consequently, if recapitulated in human patients, the repeated siRNAs can silence genes bearing complementary sequences and affect gene expression. We found that overall, there are 993 genes bearing 6 CTG or CAG-repeats in the human genome. Accordingly, we tested whether genes bearing endogenous 6 CTG/CAG repeats generally changed in expression in the patients’ transcriptome. In the DM1 tibialis samples, there are 171 differentially expressed genes containing 6 CTG/CAG repeats. These genes discriminate the DM1 patients from unaffected individuals (Figure 2A; Supplementary Figure S1). These genes are significantly enriched among the differentially expressed genes in DM1, but unexpectedly both in the up and downregulated genes (Figures 2A,B). Moreover, gene groups that carry either four to seven CTG/CAG repeats and are therefore expected targets for the CUG repeat-derived siRNAs, exhibit a significant change in expression in DM1. Specifically, we found that the expression of certain CUG/CAG-bearing genes is correlated with DM1 disease phenotypes and may predict muscle impairment (Figures 2C,D). However, genes bearing consecutive repeats of other trinucleotide pathogenic combinations (e.g., CGG) were not differentially expressed in DM1 patients (Figure 2B).

### Genes bearing CTG/CAG-repeats are differentially expressed across brain, heart, and congenital DM1 biceps biopsies

**FIGURE 3:**
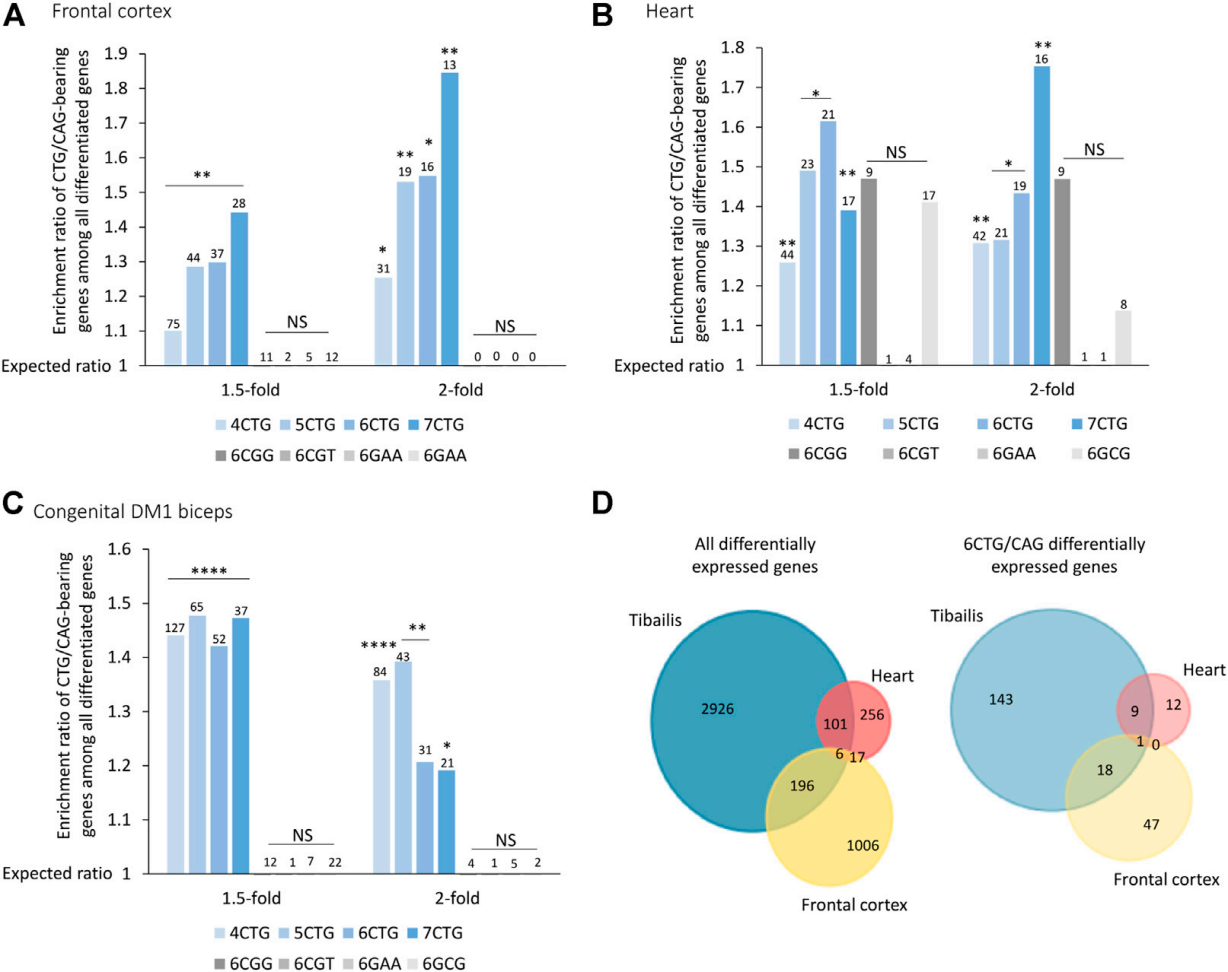
Genes bearing CTG/CAG-repeats are differentially expressed across frontal cortex **(A)**, heart **(B),** and congenital DM1 biceps **(C)** biopsies. Bar plot presents ratio of genes bearing CTG/CAG-repeats that are differentially expressed in DM1 samples divided by the expected number derived from the prevalence of those genes in the respective tissue. Different fold-change thresholds and lengths of consecutive CTG/CAG repeats are presented. Additional combinations of pathogenic repeats (6CGG, 6CGT, 6GAA, and 6GCG) are depicted in gray. The number of genes differentially expressed for each group is indicated above the corresponding bar. **(D)** Venn diagram showing overlap of all differentially expressed genes and only 6CTG/CAG-containing genes in 1,2 or three DM1 tissues. *0.05; **0.01; ****0.0001.

Our model (Qawasmi et al., 2019) suggests that while gene expression varies across tissues, in general, tissues expressing expanded CTG repeats may exhibit changes in expression levels of genes bearing endogenous CTG/CAG repeats. To investigate this, we analyzed previously published data of 21 adult frontal cortex samples (Otero et al., 2021), and nine heart autopsy samples (Wang et al., 2019) from DM1 patients (Supplementary Tables S2, S3). The CTG/CAG-bearing genes were significantly differentially expressed between DM1 patients and unaffected controls, in brain and heart (Figures 3A,B). Similar to the data from the tibialis biopsies, this enrichment was specific for CTG/CAG repeats and insignificant for other repeats.

DM1 is classified into subtypes according to the age of onset. Adult-onset DM1 is characterized by accumulative damage and slow progression over the years. On contrary, congenital DM1 (CDM1), the most severe form of the disease, is characterized by early prenatal onset, maternal inheritance, and distinct symptoms at birth (Lanni and Pearson, 2019). This discrepancy suggests a distinct molecular pathogenic mechanism of CDM1, which we believe is driven by maternal inheritance of repeat-derived siRNAs (Braun et al., 2022). CDM1 is also characterized by exceptionally long CTG repeats. We analyzed RNA-seq data from biceps biopsies of three CDM1 patients aged 3–15 months old (Thomas et al., 2017) (Supplementary Table S4). Differential expression analysis revealed a similar enrichment of CTG/CAG-bearing genes in all fold-change thresholds, despite the young age of the patients (Figure 3C).

Next, we tested if despite the different tissues, genes tend to be similarly affected. We found that 20% of the differentially expressed genes bearing 6CTG/CAG repeats in tibialis muscles of DM1 patients overlap with at least one more cohort, compared with only 9% of remaining differentiated genes (*p*-value = 0.002) (Figure 3D).

### The regulatory network of the DM1 gene expression signature

**FIGURE 4:**
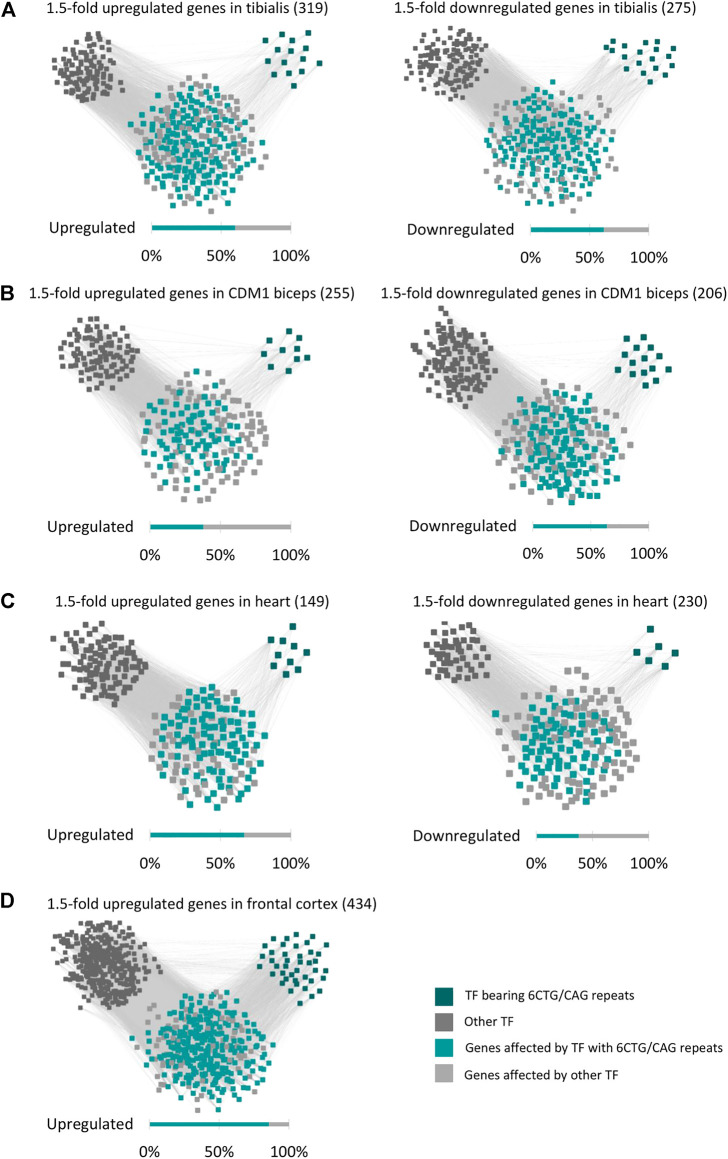
The regulatory network of the DM1 gene signature. Gene-transcription factor regulatory network of upregulated and downregulated genes in DM1 tibialis **(A)**, CDM1 biceps **(B),** DM1 heart **(C),** and DM1 frontal cortex **(D)**. Dark green squares represent transcription factors bearing 6CTG/CAG repeats and dark gray squares represent the remaining transcription factors. Cyan nodes stand for genes targeted by transcription factors bearing 6CTG/CAG repeats, light gray nodes stand for the remaining genes.

The differentially expressed genes in DM1 patients are enriched for genes bearing endogenous CUG/CAG-repeats, that may be targeted by CAG/CUG siRNAs (Qawasmi et al., 2019; Braun et al., 2022). However, these genes constitute a small part of the DM1 transcriptome. It is expected that the remaining differentially expressed genes are regulated by various transcription factors (TFs) and microRNAs. Thus, we searched for *Cis*-regulatory elements (CREs) that are over-represented in the up- or downregulated genes. Using ChEA3 (Keenan et al., 2019) we analyzed the 1.5-fold up and downregulated genes for shared transcription factor binding sites (Supplementary Table S5). Out of the 1,632 TFs that were analyzed, the binding sites (BS) of 200 are overrepresented in the regulated genes (FDR-adjusted *p*-value < 0.01). Out of these, a significant number (58, *p*-value = 3.11*10^−5^) of highly ranked (as calculated by a Kolmogorov-Smirnov test, Supplementary Figure S3) TFs are potential targets of the repeat-derived siRNAs as they contain endogenous 6CUG/CAG repeats (Supplementary Figure S4). These TFs, with 6CUG/CAG repeats, are predicted [using ChEA3 (Keenan et al., 2019)] to directly regulate half of the differently expressed genes in DM1 tibialis muscle, both up and downregulated (Figure 4A).

The significant enrichment of TFs bearing repeats was specific only to CUG/CAG repeats (as in DM1) and not to other pathogenic repeats that we tested as control such as CGT and CGG. Analysis of TFs that bear 6 CUG/CAG repeats shows that they play a key role in regulating pathways that are linked with the disease symptoms (Table 1). For example, the TFs: RUNX2, TSHZ3, and GLI-1 that bear 6 CUG/CAG repeats and were enriched for the upregulated genes, significantly regulate genes associated with extracellular matrix organization (RUNX2 and TSHZ3, FDR adjusted *p*-values: 5.7*10^−6^ and 0.0325, respectively) and collagen biosynthesis (GLI-1, FDR adjusted *p*-value = 2.7*10^−18^), pathways that were accordingly found to be upregulated in DM1 samples. We repeated this analysis for the CDM1, Heart, and Brain cohorts and demonstrated similar results (Figures 4B–D). To note, the TF enrichment analysis for the downregulated genes in DM1 Brain biopsies yielded only nine TFs, affecting 33 genes out of 211 downregulated genes. Among these, one TF contained 6CUG/CAG repeats, affecting 13 genes. Importantly, analysis of the upregulated genes in DM1 including 6CUG/CAG genes (Figure 2A) shows that most of those genes are governed by TFs containing 6CUG/CAG repeats.

### TFs bearing 6CTG/CAG repeats are overrepresented in tissues affected in DM1 patients

As mentioned above, the impact of the repeats is not expected to be specifically localized to a single tissue. The repeats are transcribed in different cell types, and the disease manifestation is multi-systemic. Altered expression of transcription factors can be detrimental to the homeostasis of normal cellular function and may damage tissues. Consequently, we analyzed the expression of the TFs bearing 6CTG/CAG repeats in various systems. We found [using GeneAnalytics (Fuchs et al., 2016)] that TFs with endogenous 6CTG/CAG repeats show the strongest over-expression signal (Table 2; Supplementary Figure S5) in the nervous systems, sensory organs, and the musculoskeletal systems. These systems correlatively exhibit the most prominent and widespread DM1 symptoms: CNS dysfunction, cataracts, hearing impairment, myotonia, and distal muscle weakness, respectively.

**TABLE 2:** Enrichment of transcription factors bearing 6CTG/CAG repeats in body systems. TFs with CTG/CAG repeats are significantly expressed in tissues presenting symptoms associated with DM1. System scores and *p*-values following FDR are presented.

System	Score	*p*-value	Symptoms
Nervous system	662	2.25E-06	Cognitive impairment, personality disturbance, mental retardation
Sensory organs	6.88	9.15E-05	Early-onset cataracts, hearing impairments
Musculoskeletal system	6.87	1.66E-07	Myotonia, progressive muscle weakness
Urinary system	4.73	0.0004	Renal dysfunction
Hematopoietic system	4.52	0.0022	IgG and IgM hypogammaglobulinemia
Integumentary system	4.46	4.79E-06	Premature frontal balding
Reproductive system	4.26	0.0001	Complications of pregnancy
Gastrointestinal system	4.07	0.0001	Irritable bowel symptoms, dysphagia
Endocrine system	3.11	0.0004	Glucose intolerance, hypogonadism
Early embryonic tissues	2.35	0.18	
Extraembryonic tissues	2.19	0.003	
Respiratory system	1.98	0.005	
Cardiovascular system	1.97	0.039	Conduction disturbances
Hepatobiliary system	1.95	0.002	Gallstones, fatty river

### Overlapping microRNA gene sets were identified for upregulated genes in DM1 patients

**FIGURE 5:**
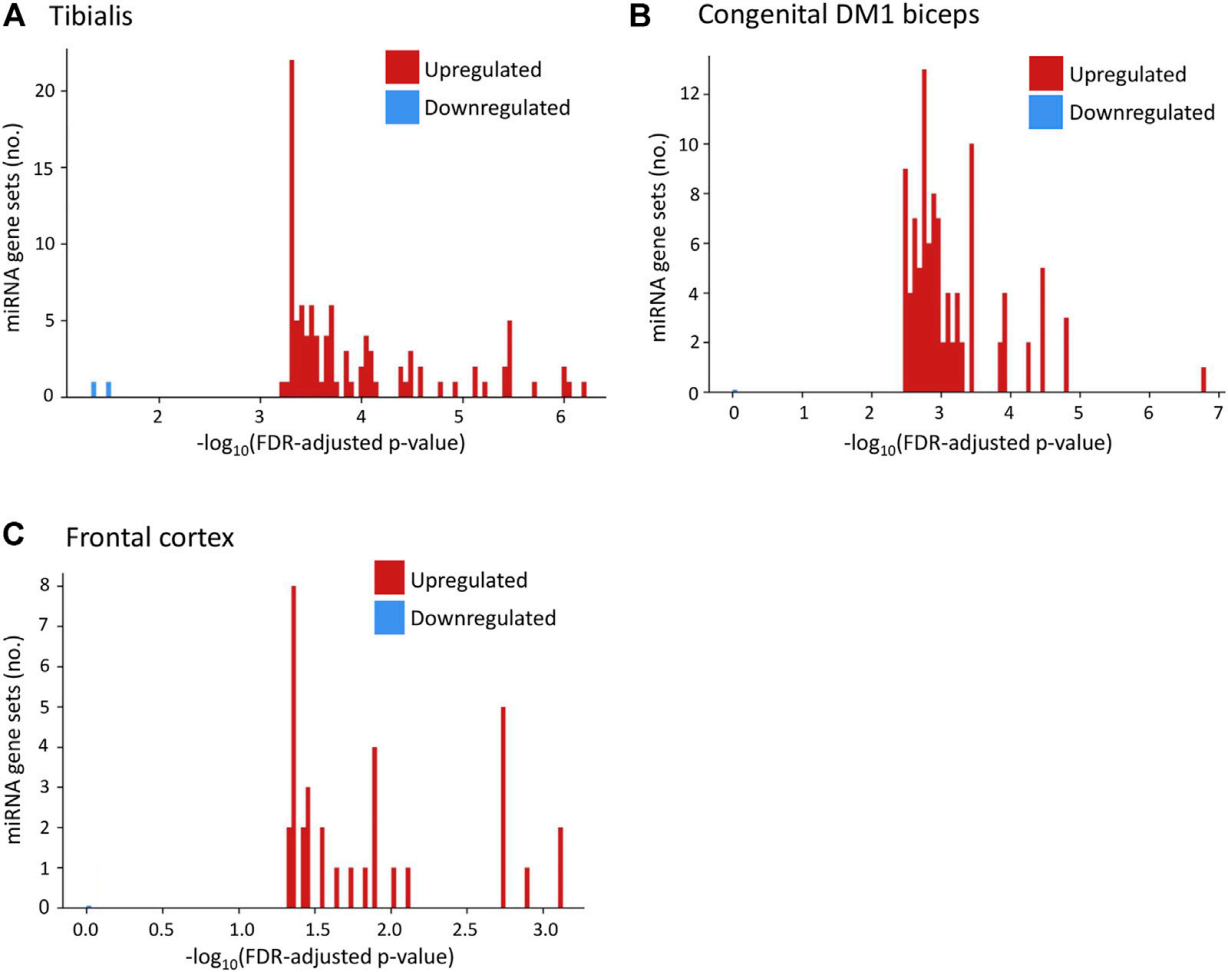
Upregulated genes in DM1 tissues are predicted targets of multiple miRNAs. Histograms present the number of miRNA gene sets enriched for upregulated (red) and downregulated (blue) genes in DM1 by their value of [−log10(*p*-value)] for tibialis **(A)**, congenital DM1 biceps **(B),** and frontal cortex **(C)** biopsies.

Using GSEA (Subramanian et al., 2005) we found (Figure 5A; Supplementary Table S6) that the upregulated genes in Tibialis DM1 muscle biopsies are predicted targets of over than 100 miRNAs (FDR adjusted *p*-values range from 5.99*10^−7^ to 5.81*10^−4^). In contrary, the group of downregulated genes are not predicted to be targeted by any miRNA (*p*-value > 0.01). Similarly, upregulated genes in brain DM1 tissues (Otero et al., 2021) were enriched for 34 miRNAs whereas the downregulated genes were not enriched (Figure 5B). Assessment of congenital DM1 biceps biopsies (Thomas et al., 2017) recapitulated this phenomenon, with over 100 significant miRNAs predicted to regulate the upregulated genes and 0 for the downregulated (Figure 5C). Recent studies also directly show that miRNAs are downregulated in DM1 patients’ hearts (Kalsotra et al., 2014; Koehorst et al., 2020). Summarizing, the upregulated genes in all tissues are predicted miRNA targets, as opposed to the downregulated genes, contrary to expectations.

## Discussion

**FIGURE 6:**
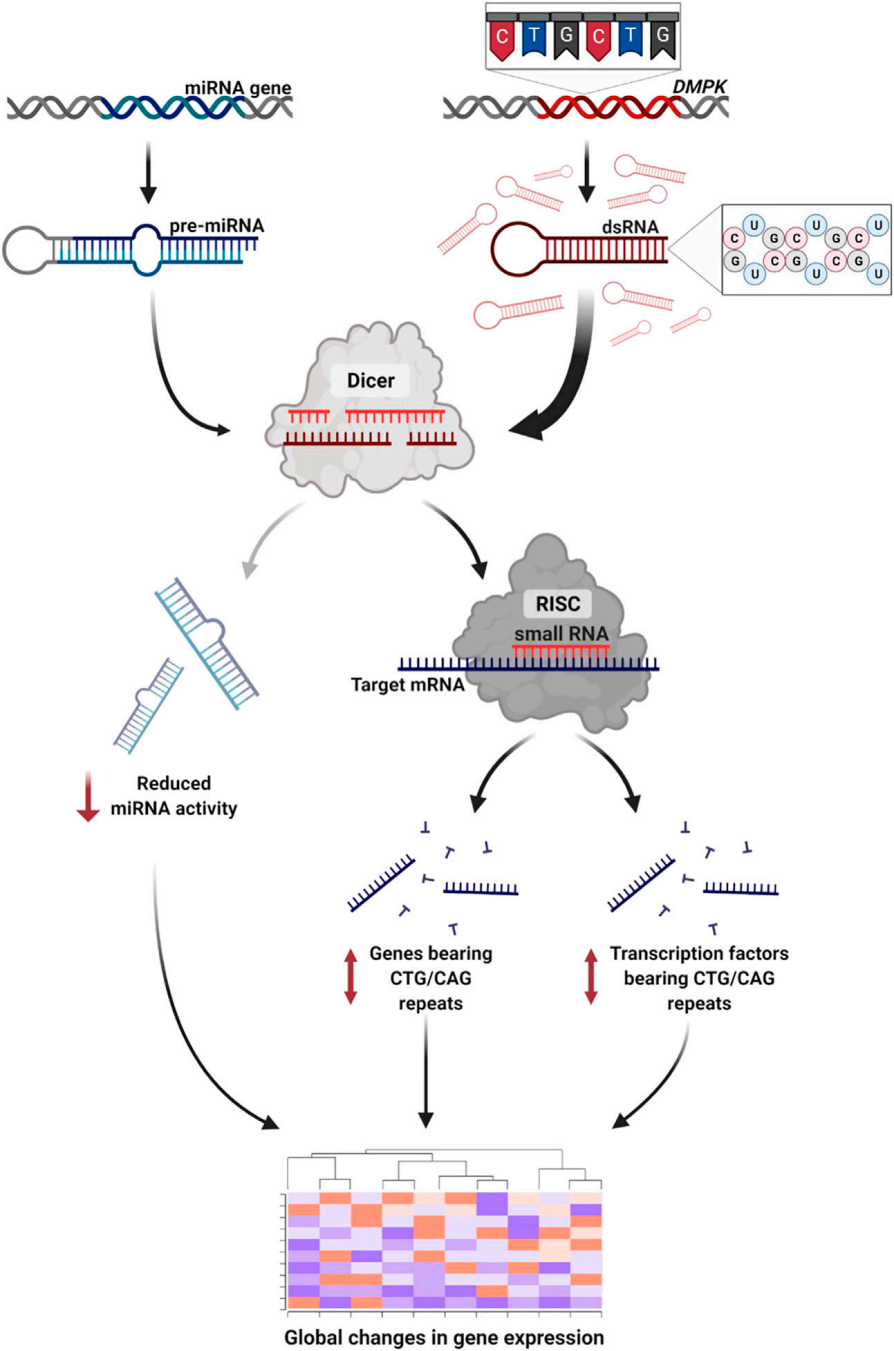
RNA interference mediates toxicity in DM1. The expanded repeat sequence is transcribed to toxic RNA that forms stem-loop structures. The double-stranded RNA is subsequently cleaved by Dicer to short-interfering RNA. These activate the RNAi silencing pathway triggering global changes in gene expression through three pathways: 1) Disrupted miRNA activity due to reduced number of miRNAs reaching maturity. 2) Altered expression of genes bearing CTG/CAG repeats. 3) Altered expression of transcription factors bearing CTG/CAG repeats. Created with BioRender.com.

In this small-scale metanalysis we investigate the transcriptional changes in DM1 patients across tissues. We show that the pathophysiology of the disease is reflected and can be predicted by differential gene expression across patients. We suggest that the expression level of many genes can be at least partly explained by the direct and indirect activity of TFs, microRNAs and CUG repeat-derived siRNAs. Specifically, we found altered expression in genes and TFs containing endogenous CTG/CAG repeats (Figure 6). The effect on the TF triggers changes in the TF’s downstream target genes which comprise a significant part of the differentially expressed genes in patients’ biopsies.

In previous work in *C. elegans*, we demonstrated that repeat-derived siRNAs can target transcripts bearing endogenous CUG/CAG repeats and induce disease symptoms through the RNAi machinery (Qawasmi et al., 2019; Braun et al., 2021; Braun et al., 2022). Knockdown of key components of the RNAi pathway resulted in the rescue of the disease phenotype. The data presented here from human DM1 patients supports the role of the RNAi machinery in DM1 pathogenesis. To note, the CUG/CAG-bearing genes were also enriched among the upregulated genes. We hypothesize this may be a result of genetic compensation through a positive feedback loop that upregulates the degraded genes (El-Brolosy et al., 2019).

While several mechanisms were suggested in repeat diseases (Miller, 2000; Krol et al., 2007; Klein et al., 2011; Zu et al., 2011; Donnelly et al., 2013; Fernandez-Costa et al., 2013; Xu et al., 2013), none of them can explain all the clinical effects and the gene expression signature. Sequestration of MBNL proteins by the expanded repeats and subsequent dysregulation of splicing is considered a major pathogenic mechanism. Du et al. (2010) found significant overlap of differential gene expression between MBNL1 knockout and CTG repeat mice. However, in DM1 patient biopsies, less than 4% of the significantly differentiated genes showed dysregulated splicing, including known MBNL targets (Supplementary Figure S6).

The global changes in miRNA activity can be a result of temporal and physical disturbance in the maturation process lead by Dicer, caused by competition between the CUG/CAG repeats and the other microRNAs. The pattern of the upregulated genes in DM1 samples suggests a malfunction in the miRNA regulatory pathway. This is well supported by findings of downregulated miRNAs in *Drosophila* models of CTG expansions (Fernandez-Costa et al., 2013). Juźwik et al. (2019) reviewed numerous papers investigating repeat expansion disorders and concluded that miRNAs were predominantly downregulated in both myotonic dystrophies and Huntington’s disease. These findings, together with splicing variations resulting from MBNL sequestration, may account for the broad DM1 gene expression signature. We speculate that the processing of the repeat-derived siRNAs by Dicer may compete with the endogenous miRNA processing over limited resources, resulting in reduced miRNA activity. This theory necessitates further validation in animal and cellular DM1 models.

DM1 is a multisystemic disease, however, the link between the affected systems in DM1 is unclear. To date, a global mechanism that explains the various manifestations remains uncovered. The common pathological driver is the expansion of the repeats. We suggest a molecular mechanism, linking the expanded repeats to a disturbed gene expression signature, through the RNAi machinery. Consequently, every organ expressing the expanded repeats and an intact RNAi machinery may be affected. Genes are differentially expressed in distinct tissues, varying in roles and importance to normal cell function. Hence, this may explain why some tissues are more impaired than others, and the wide divergence of symptoms from muscle dysfunction to cataract. We found an enrichment of CTG/CAG-bearing genes in distinct DM1 patient organs, thus further supporting our mechanism.

Future research should investigate specific dysregulated genes that may be related to unexplained disease symptoms. For example, COL5A1 and NHS, both genes bearing endogenous CTG/CAG repeats, therefore potential silencing targets of the toxic siRNAs, are known causes of cataract (Shiels and Hejtmancik, 2015). Cataract is the most prevalent symptom of DM1, affecting almost all patients. ATF4 is a 6CTG -bearing transcription factor that was highly dysregulated in DM1 patients. It was recently established as an important factor of maintenance of long-term memory (Smith et al., 2020). Memory impairment is also described as a characteristic DM1 symptom (Rubinsztein et al., 1997; D’Angelo and Bresolin, 2006). Additionally, enrichment analysis of differentially expressed genes identified pathways associated with fibrosis. DM1 patients with end-stage disease are prone to developing fibrosis through an unknown mechanism (Gagnon et al., 2010; Meola and Cardani, 2015; De Antonio et al., 2016). The genes showcased here represent a small part of the puzzle; further research and validation is essential.

To conclude, we present evidence that DM1 patients exhibit extensive dysregulation of genes bearing endogenous CTG/CAG repeats. These data support the central role played by the RNAi machinery of mediating toxicity in repeat-based disorders. This mechanism offers a potential novel therapeutic approach to overturn the DM1 gene signature by targeting the RNAi machinery, pending further validation in mammalian models.

## Data Availability

The datasets presented in this study can be found in online repositories. The names of the repository/repositories and accession number(s) can be found below: GEO repository under accession numbers: GSE97806, GSE157428, and GSE86356. (https://www.ncbi.nlm.nih.gov/geo/query/acc.cgi?acc=GSE86356, https://www.ncbi.nlm.nih.gov/geo/query/acc.cgi?acc=GSE157428, https://www.ncbi.nlm.nih.gov/geo/query/acc.cgi?acc=GSE97806).
