# Supplementary material for: Transcriptome changes in DM1 patients’ tissues are governed by the RNA interference pathway
Source: Front Mol Biosci. 2022 Aug 19;9:955753. doi: 10.3389/fmolb.2022.955753 (PMC9437208; doi:10.3389/fmolb.2022.955753)
Supplement: Supplementary file 7 [file DataSheet1.docx]

Supplementary Material

# Supplementary Figures and Tables

Supplemental Table S1: Expression results of differentially expressed genes in Tibialis DM1 muscle biopsies.

Supplemental Table S2: Expression results of differentially expressed genes in Brain DM1 biopsies.

Supplemental Table S3: Expression results of differentially expressed genes in Heart DM1 biopsies.

Supplemental Table S4: Expression results of differentially expressed genes in congenital DM1 biceps muscle biopsies.

Supplemental Table S5: Gene set enrichment analysis presenting the transcription factor target gene sets for 1.5-fold upregulated and downregulated genes in DM1 samples.

Supplemental Table S6: Gene set enrichment analysis presenting top 100 regulatory miRNA target gene sets for upregulated and downregulated genes in DM1 samples.

## Supplementary Figures

**
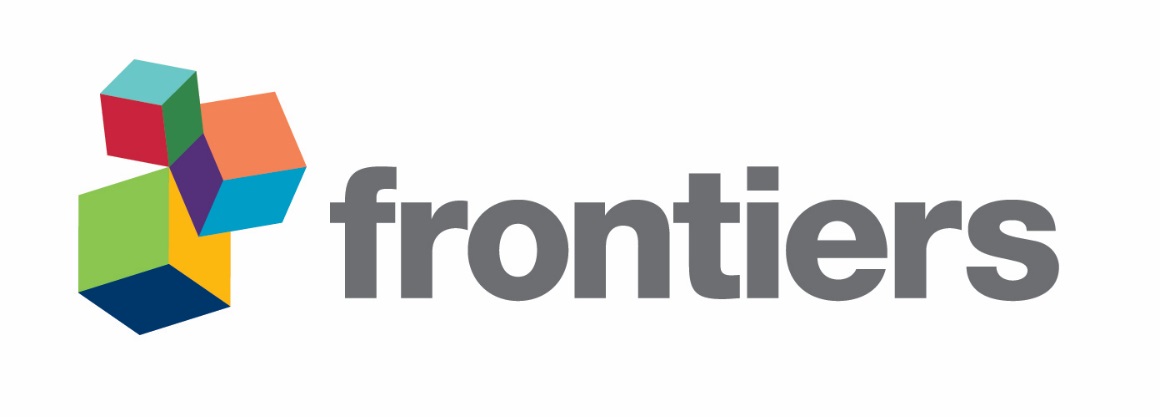
**

**
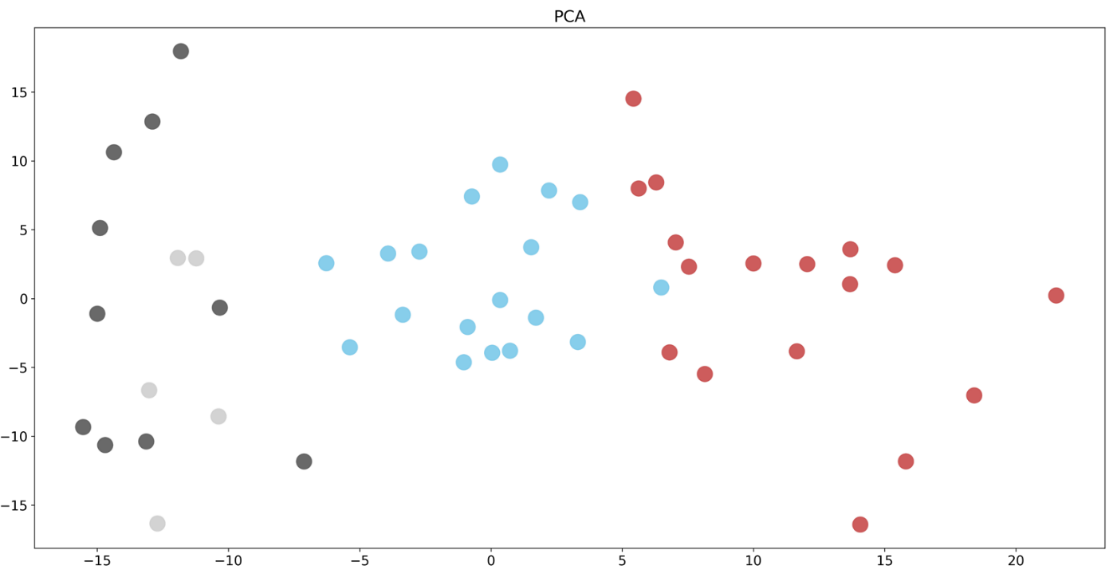
**

**Supplementary Figure 1.** PCA analysis of normalized gene expression values for genes bearing 6CTG/CAG repeats in Tibialis DM1 and healthy samples

**
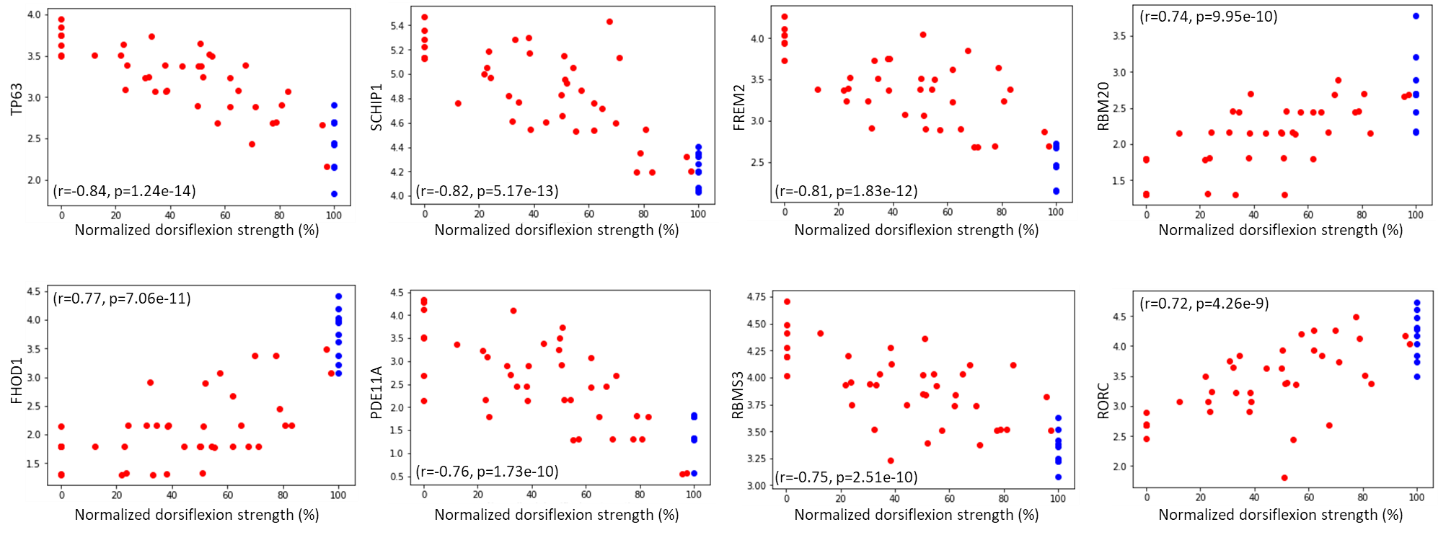
**

**Supplementary Figure 2.** Normalized dorsiflexion strength scores in correlation to expression levels of 6CTG/CAG genes


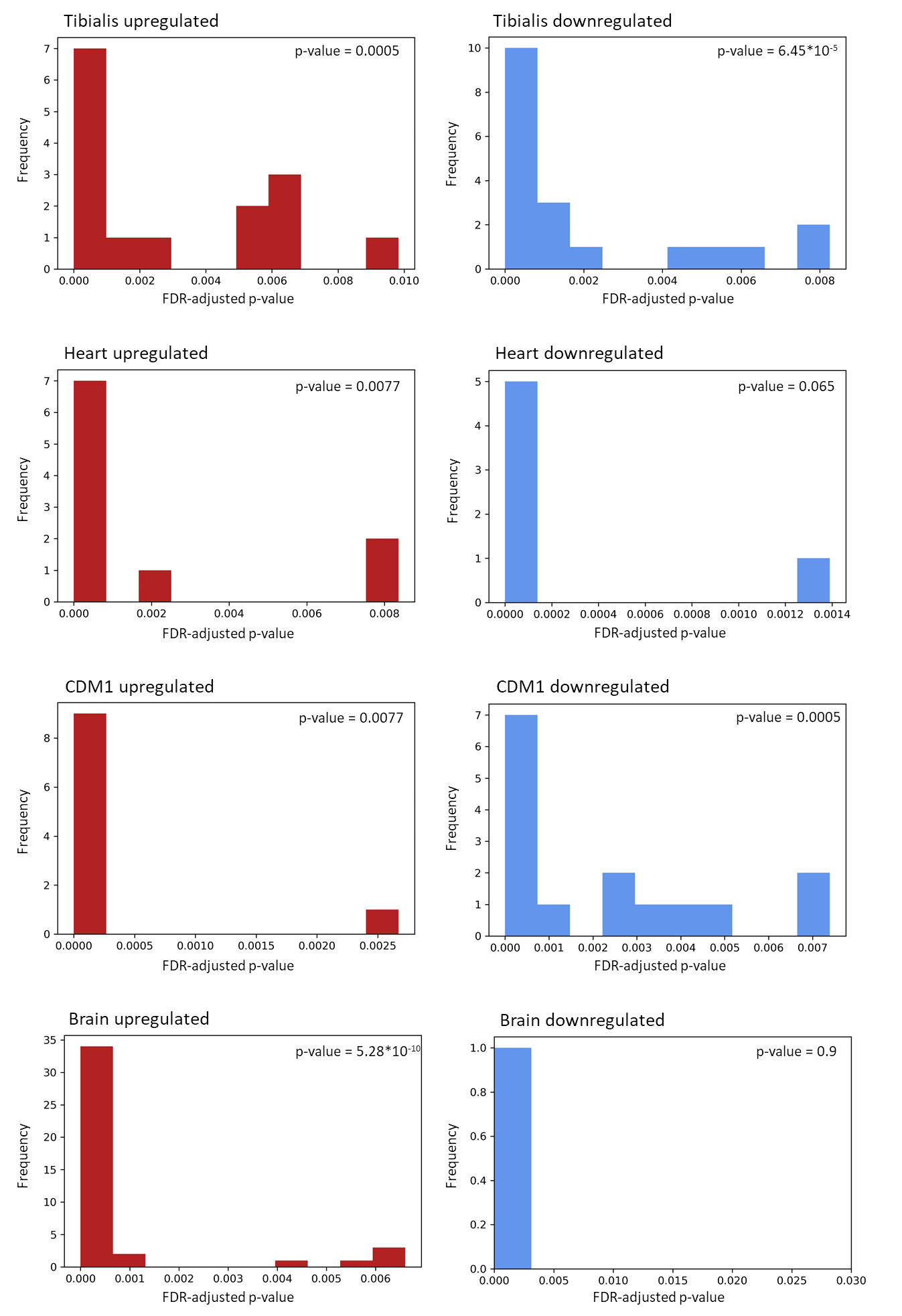


**Supplementary Figure 3.** Distribution of significantly enriched 6CTG/CAG-bearing transcription factors according to their FDR-adjusted p-values. Kolmogorov-Smirnov test values are depicted.

**
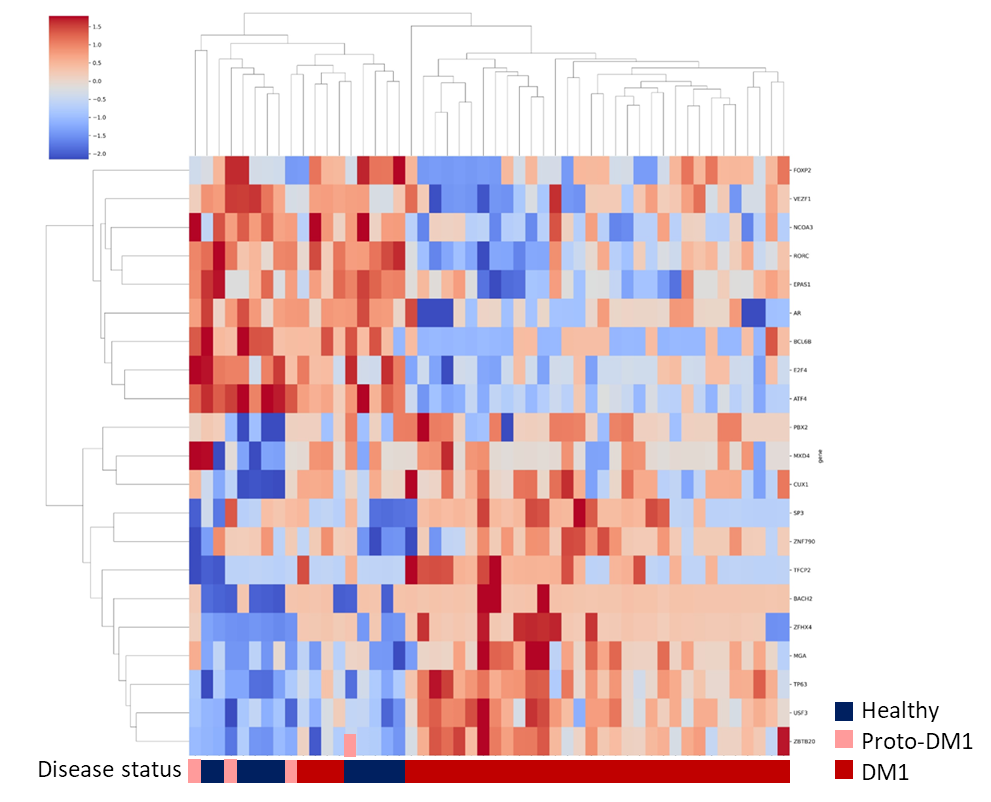
**

**Supplementary Figure 4.** Heatmap of normalized expression levels of the differentially expressed 6CTG/CAG-bearing transcription factors across Tibialis samples from 40 DM1 patients and 10 healthy individuals. The rows and columns are ordered based on hierarchical clustering (by the UPGMA method). Each row represents a single gene, and each column represents a sample. The colors indicate the normalized expression levels from high (red) to low (blue).


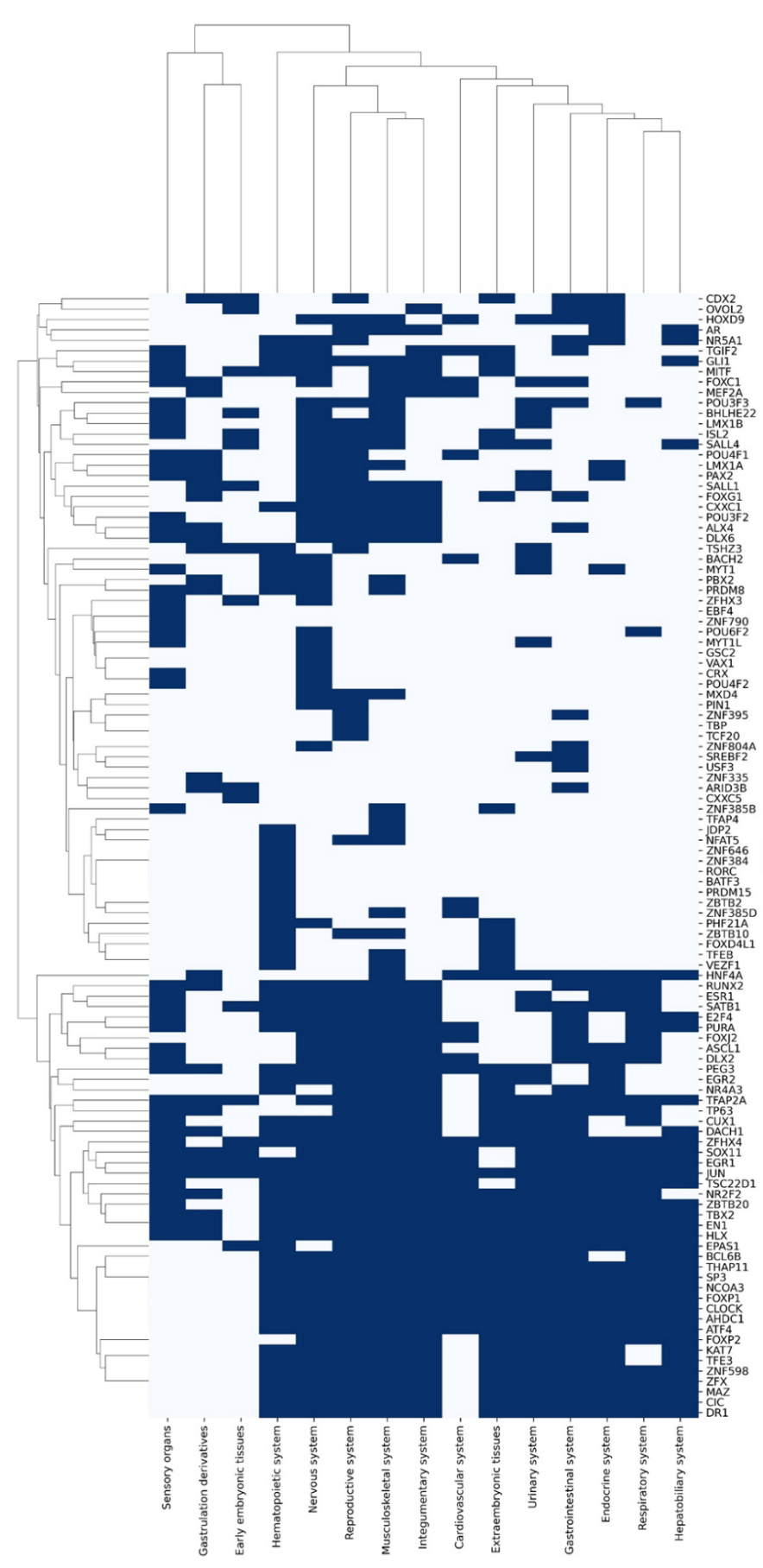


**Supplementary Figure 5.** Transcription factors bearing 6CTG/CAG repeats are enriched in disease affected systems. Each row represents a single transcription factor, each column represents a system. Dark blue indicates enriched expression (see Table 2, p-values of < 0.05 following FDR were considered significant).


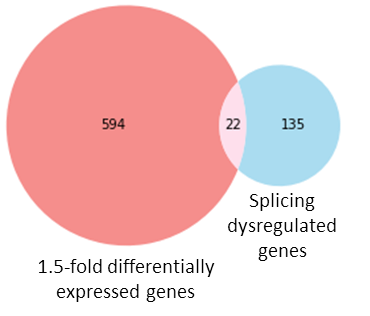


**Supplementary Figure 6.** Venn diagram showing overlap of 1.5-fold differentially expressed genes and splicing dysregulated genes in DM1 tibialis biopsies (Wang et al., 2019).
